# Epigenetic regulation of the β-catenin pathway in cadmium-induced colorectal cancer progression and chemoresistance

**DOI:** 10.3389/fcell.2026.1808500

**Published:** 2026-04-22

**Authors:** Rajkumar Prabhakaran, Ramkumar Muthu, Karthikeyan Mahendran, Rajkumar Thamarai, Venkatesh Subramanian, Sorimuthu Revathi, Balasubramanian Velramar, Shanmugapriya Dharani, Balachandran Ruthramurthy

**Affiliations:** 1 Department of Biochemistry, Karpagam Academy of Higher Education (Deemed to be University), Coimbatore, Tamil Nadu, Ethiopia; 2 Centre for Cancer Research, Karpagam Academy of Higher Education, Coimbatore, Tamil Nadu, India; 3 Department of Biotechnology, Sri Kaliswari College (Autonomous), Sivakas, Tamil Nadu, India; 4 Department of Microbiology, PSG College of Arts and Science, Coimbatore, Tamil Nadu, India; 5 Department of Respiratory Medicine, Saveetha Medical College and Hospital, Saveetha Institute of Medical and Technical Sciences (SIMATS), Saveetha University, Chennai, Tamil Nadu, India; 6 Department of Biotechnology, Manonmaniam Sundaranar University, Tirunelveli, Tamil Nadu, India; 7 Department of Emergency Medicine, Centre for Global Health Research, Saveetha Medical College and Hospital, Chennai, Tamilnadu, India; 8 Amity Institute of Biotechnology, Amity University Chhattisgarh, Raipur, Chhattisgarh, India; 9 Vicerrectoría de Investigación y Postgrado, Universidad de La Serena, La Serena, Chile; 10 Department of ECE, Adama Science and Technology University, Adama, Ethiopia

**Keywords:** colorectal cancer, epigenetic modifications, microRNA, non-coding RNA, β-catenin signaling pathway

## Abstract

Colorectal cancer (CRC) has become a global health issue, as exposure to toxic metals (lead, chromium, cadmium, aluminum, copper, arsenic, and mercury) has all been implicated in its development and progression through interference with the mechanisms of cell proliferation and death. Recent research suggests that cadmium (Cd) and other metals disrupt normal cellular homeostasis through epigenetic changes, particularly modifying the β-catenin signaling pathway, an important key regulator of cell proliferation, differentiation, and survival. This study explores and examines the molecular mechanisms of Cd-induced changes in DNA methylation, histone acetylation, and non-coding RNA, which increase β-catenin activation and translocation into the nucleus. Such atypical epigenetic alterations promote oncogene transcriptional upregulation and survival pathways to promote tumorigenesis and non-response to chemotherapy agents. Altered epigenome regulation and dysregulation of the β-catenin pathway exacerbate Cd’s oxidative stress and inflammatory capacities, which in turn lead to tumor-promoting microenvironment capabilities. The alterations uncovered with Cd exposure and epigenetic reprogramming, as well as the altered β-catenin signaling pathways, offer additional information regarding the molecular etiology of Cd-induced CRC. This review also suggests new possible treatments involving epigenetic regulators and β-catenin signaling components as exciting therapeutic approaches to address chemotherapy resistance and improve treatment outcomes for patients with colorectal cancer who have previously been exposed to heavy metals.

## Graphical Abstract

**Figure:**
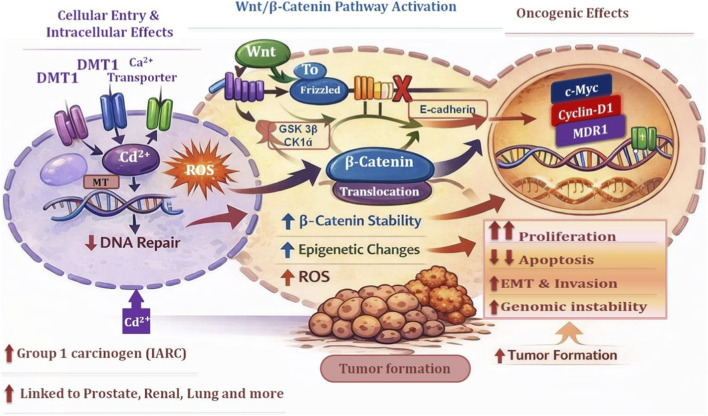

## Highlights of the study

- Toxic metals such as cadmium contribute to colorectal cancer initiation and progression.
- Metal exposure enhances tumor growth and chemoresistance.
- Epigenetic dysregulation disturbs β-catenin signaling, promoting cancer cell survival.
- Aberrant DNA methylation and histone modification active oncogenic pathways.
- Targeting epigenetic and β-catenin regulators may prove to be CRC therapy in metal-exposed patients.

## 1 Introduction

Cadmium (Cd) contamination is now an urgent worldwide concern for public health and ecology due to rapid industrialization, along with an increasing use of Cd in agriculture and environmental persistence (Charkiewicz et al., 2023; Zulfiqar et al., 2022; Joseph, 2009). Recent epidemiological and experimental research has shown that prolonged exposure to Cd is a significant factor in the carcinogenic development of colorectal cancer (CRC). CRC is one of the most frequent cancers worldwide and is one of the leading causes of morbidity and mortality from cancer, with approximately 1.9 million new cases and around 940,000 deaths from this disease alone in 2020 (Xi and Xu, 2021; Tian et al., 2022). CRC poses a considerable clinical challenge, despite the advances in early detection and therapeutic intervention. There is a range of contributing factors to the aetiology of CRC; therefore, it progresses aggressively and is often resistant to chemotherapy. Cd is an incredibly hazardous metal that has a wide range of industrial applications, including battery manufacturing, electroplating, pigments, and the manufacture of plastic products (Khan et al., 2022). It is present in the surroundings of everyday living, with people mainly consuming it through polluted foods and drinks, direct industry byproducts, and cigarette smoke. Because of its high carcinogenic potential and prolonged half-life in biology, the International Agency for Research on Cancer (IARC) has classified Cd as a human carcinogen (Briffa, et al., 2020; Rahman and Singh, 2019). There are many occupational and epidemiological studies that show a strong correlation between Cd exposure and the incidence of various cancers, including CRC (Zhang et al., 2024; Kavalukas et al., 2025). Additionally, several studies have shown there are significantly elevated plasma levels of Cd in CRC patients (Mahmood et al., 2022). Also, studies *in vitro* have shown that Cd can cause the malignant transformation of human colon epithelial cells (Lu et al., 2015).

Cd can lead to cancer through several different biological processes: exposure to oxidative stress, chronic inflammation, and changes to DNA; disrupt normal cell growth and/or promote cancer formation. In addition, recently, new approaches have been used to develop methods of studying the relationship between Cd exposure and the effects on human health and to identify genetically relevant targets for intervention to reduce CRC. Therefore, significant advancements have been made over the past few years to further investigate the link between Cd exposure and CRC initiation and progression, the molecular pathways that are involved in the initiation of CRC, and the potential influence of Cd on drug resistance to CRC therapies. However, although there continues to be a strong interest in the molecular pathways that may play a role in the action of Cd, as well as understanding the different types of data available to study biological effects at the DNA level *in vitro*, there is still no clear understanding of how Cd can produce CRC by causing epigenetic changes, especially when using current methods.

CRC is influenced by multiple interacting factors, including genetic predispositions, environmental carcinogens, and lifestyle behaviors like diet, obesity, and tobacco usage. Nowadays, researchers concede the significant role of epigenetic alterations, DNA methylation, modification of histones, and dysregulation of non-coding RNAs as mediators of the relationship between environmental factors and heritable changes to gene expression without any alteration in the DNA sequence. Epigenetic mechanisms, therefore, are important participants in CRC induction, promotion, and metastasis, as well as resistance to drug therapy (Cao et al., 2024; Prabhakaran et al., 2026a). Cd in the environment can greatly affect the epigenetic landscape to enhance oncogenic signaling pathways and inhibit tumor suppressor signaling pathways. One of the most commonly altered pathways in CRC is the canonical Wnt/β-catenin pathway, which is critical for the maintenance of intestinal stem cells and their renewal for continued homeostasis of a given intestinal tissue structure. The regulation of β-catenin to ensure stability within cells typically occurs through the regulation of cytoplasmic destruction complexes; however, mutation of the APC gene or aberrant stabilization of β-catenin leads to the accumulation of β-catenin in the nucleus, where β-catenin acts as a cofactor for transcriptional activation of genes regulating proliferation, survival, invasion/migration, and stem cell phenotype. Interestingly, constitutive activation of Wnt and β-catenin signaling is present not only in the majority of CRC but also has been shown to correlate with poor tumor aggressiveness, metastasis, and drug resistance (Novoa Diaz et al., 2022; Wu et al., 2025).

Genetic changes to the Wnt/β-catenin signaling pathway have been studied extensively in CRC; however, much less is known about the effects of environmental carcinogens, like Cd, on this signaling pathway and how epigenetics may mediate these effects. Emerging studies indicate that Cd exposure produces systemic epigenetic changes, including aberrant DNA methylation patterns, the dysregulation of microRNAs, and the dysregulation of other classes of non-coding RNAs that ultimately affect β-catenin signaling and downstream oncogenic molecular networks. It is important to point out that these epigenetically mediated changes have been implicated in the initiation and progression of CRC as well as the emergence of chemotherapy resistance, which represents a significant clinical challenge for patients with CRC (Marei, 2025). Recent findings illuminate a complex interplay between Cd-induced epigenetic alterations, nutritional components, gut microbiota, and miRNA-based regulation of the Wnt/β-catenin signaling pathway. As a result, miRNAs targeting β-catenin have become of great interest as potential alternative approaches to treating diseases caused by dysregulation of β-catenin through use of direct inhibitors with severe side effects and poor bioavailability. In addition, cutting-edge nanomedicine delivery systems, especially those made from marine resources; provide additional options to improve the specificity, stability, and clinical efficacy of therapeutic agents (Muthu et al., 2025; Prabhakaran et al., 2024; Prabhakaran et al., 2026b).

Despite increased evidence elucidating the association between Cd exposure and the development of CRC, the molecular pathways by which Cd contributes to CRC have so far not been fully elucidated. Especially, the role of Cd-induced epigenetic alterations in regulating key tumor signaling pathways has not yet been completely integrated into the current understanding. One of the pathways potentially impacted by Cd-induced epigenetic changes is the Wnt/β-catenin signaling cascade, a vital pathway in maintaining intestinal homeostasis. Moreover, as it acts as a key factor involved in the initiation, progression, metastasis, and chemoresistance of tumors, this pathway is frequently dysregulated in CRC. Recent reports have also indicated that Cd exposure may modify epigenetic mechanisms such as DNA methylation, histone modification, and dysregulation of non-coding RNAs through the influence of β-catenin signaling and associated transcriptional programs to affect tumor proliferation and therapeutic resistance. Thus, the primary objective of this review is to analyze research concerning the epigenetic regulation of Wnt/β-catenin signaling in CRC associated with Cd exposure and to evaluate the potential role that Cd-mediated epigenetic alterations may play in the regulation of β-catenin signaling and, consequently, in the development of CRC and chemoresistance. Furthermore, by integrating data derived from experimental and clinical studies on Cd exposure, this study aims to enhance our understanding of the mechanisms by which environmental exposure influences epigenetic regulation and cancer signaling pathways in CRC, as well as to identify epigenetic targets for possible future therapeutic options.

## 2 Methodology

This narrative review was conducted by performing a systematic literature search in order to find studies related to the epigenetic modifications of the β-catenin signaling pathway in Cd-induced CRC and chemoresistance. Electronic databases were searched for studies published in the time period 2020–2026 through PubMed, Scopus, and Web of Science. The main aim of this narrative review was to evaluate publications that have been published from 2000 until 2026; however, some older foundation studies were used as necessary to give an overview of the background. A combination of keywords and Boolean operators was used to retrieve relevant articles, including “cadmium,” “colorectal cancer,” “β-catenin,” “Wnt/β-catenin signaling,” “epigenetic regulation,” “DNA methylation,” “histone modification,” “microRNA,” “chemoresistance,” and “heavy metal toxicity.” Selection criteria included relevance to Cd exposure, epigenetic mechanisms, the Wnt/β-catenin signaling pathway, colorectal cancer development, and chemoresistance. We applied our selection criteria to both experimental studies (*in vitro* and *in vivo*) and clinical studies. Reference lists from the selected articles were hand-searched for additional articles that met criteria; only articles published in English and available in full-text form were included in our review. Most review articles were used as background references; most original research articles were prioritized in the mechanistic discussion of epigenetic changes caused by Cd and their contribution to the progression of CRC and chemoresistance.

## 3 Cadmium exposure and colorectal carcinogenesis

Cd is a toxic, heavy, nonessential metal that has been classified as a human carcinogen by multiple international health organizations because of its ubiquity and its potential to disrupt normal cell function. Sources of Cd contamination include industrial emissions (company smoke), cigarette smoke, drinking polluted water, and eating foods that are contaminated with Cd. The biological effects of Cd accumulate in human tissues over time, increasing the likelihood of developing cancers throughout the body. Although there is a great deal of scientific literature on the carcinogenic effects of Cd on lung and prostate cancers, there is now a significant body of evidence linking Cd to CRC as an important factor in developing and maintaining this disease. More research is needed to understand how Cd plays a role in the mechanisms that result in the occurrence and progression of CRC. Epidemiological and experimental research reveals that prolonged exposure to Cd may be associated with an increased incidence of CRC. This may be caused by an induction of oxidative stress as well as the disruption of DNA repair processes or the alteration of signaling pathways that regulate cell proliferation or cell survival due to exposure to toxic metals. Accumulation of Cd in the gastrointestinal tract after ingestion makes the colon an appropriate target for Cd toxicity. Furthermore, systematic reviews have shown that the potential for Cd to stimulate cell survival and growth may also contribute to an elevated risk for CRC based upon additional toxic metals in the exposure profile (Bonfiglio et al., 2024). Another significant method Cd influences the development of cancer is through creating oxidative stress from the overproduction of reactive oxygen species (ROS). Cd causes an imbalance in the antioxidant defense systems compared to the pro-oxidant forces, which may lead to damage to lipids, proteins, and DNA in the colonic epithelial cell compartment. The overall oxidative load caused by this oxidative stress then contributes to genomic instability, which is one of the key indicators of cancer development.

The systematic reviews have also confirmed how the oxidative stress (redox stress) and inflammatory processes caused by Cd are contributing factors in the processes of carcinogenesis, as well as noting that the overproduction of ROS and the activation of pro-inflammatory signaling transcription factors, such as NF-κB, may be instrumental in creating a long-lasting injury to the cells and causing neoplastic transformation. Chronic exposure to ROS can cause activation of stress-dependent signaling pathways like p38 MAPK and downstream effectors, leading to survival signals for damaged cells, allowing them to escape apoptosis and continue cell proliferation. Table 1 summarizes the Cd-induced mechanisms in CRC across oxidative stress, Wnt/β-catenin signaling, and invasion pathways, with evidence hierarchy from human tissue analyses to *in vitro* studies (Bailey et al., 2025; Zhang et al., 2024).

**TABLE 1:** Mechanism of Cd-induced colorectal carcinogenesis.

Aspect	Mechanism/Effect	Key pathways/Markers	Evidence type	Key findings	References
Oxidative stress	Cd elevates ROS *via* NOX1 activation, leading to DNA damage (e.g., 8-OHdG).	NOX1↑, SOD1↓, SOD2↓, CAT↓, 8-OHdG↑	Animal (mouse)	Cd intensified ROS in CC tumors, promoting proliferation.	Zhang et al., 2024; Chakraborty et al., 2010
Wnt/β-catenin signaling	Cd disrupts Wnt secretion, stabilizes β-catenin by inhibiting GSK3β.	β-catenin↑, p-GSK3β↑	Animal/*In vitro*	Cd amplified β-catenin accumulation in CC cells.	Zhang et al., 2024; Chakraborty et al., 2010
Cell proliferation	Enhanced tumor growth and fluorescence *in vivo*.	Proliferation markers ↑	Animal (mouse)	Cd-O2/N2 gas boosted CC cell proliferation (p < 0.001).	Zhang et al. (2024)
Invasion/Migration	Upregulates MMP-2 for remodeling and metastasis.	MMP-2↑	Animal/*In vitro*	Cd facilitated tumor restructuring and migration.	Naji et al. (2019)
Inflammation	Dual effect: Mucosa suppression, tumor promotion *via* macrophages.	Iba1, MMP-9, COX-2 altered	Animal (mouse)	Cd heightened Iba1 in tumors but reduced in mucosa.	Zhang et al. (2024)
Tissue element levels	No Cd difference; Cu↑, Se↑, Mg↑ in malignant tissue.	Cu (1.10 vs 0.64 μg/g), Se (0.12 vs 0.05)	Human (n = 25)	Disturbed Zn/Cu and Ca/Mg ratios in CRC tissue.	Klimczak et al. (2016)
ROS-p38-COX-2	Low-dose Cd promotes migration *via* ROS-dependent pathway.	ROS-p38-COX-2-PGE2, Akt	*In vitro* (HT-29)	Cd increased migration; blocked by NAC or p38 inhibitor.	Klimczak et al. (2016)
MAPK/VEGF Angiogenesis	Dose-dependent: low Cd boosts angiogenesis *via* ERK/JNK/p38.	MAPK (ERK, JNK, p38), VEGF↑	Review/*In vitro*	Low Cd enhances tumor angiogenesis.	Xing et al., 2024; Wei et al., 2017
Apoptosis/DNA damage	Cd induces oxidative stress, DNA damage response, inhibits apoptosis.	BCL2-BAX, oxidative stress	Review	Cd linked to apoptosis dysregulation in cancers.	Bailey et al., 2025; Yu et al., 2024
Cytotoxicity in CRC cells	Dose-dependent viability loss in HT-29 cells (≥1 μg/mL Cd).	Cell viability ↓, morphology changes	*In vitro* (HT-29)	Cd cytotoxic at low concentrations in CRC lines.	Iftode et al. (2021)

↑↓, significantly increased/decreased (p < 0.05); ROS, Reactive oxygen species; 8-OHdG = 8-hydroxydeoxyguanosine (oxidative DNA damage marker); NOX1, NADPH oxidase 1; SOD, Superoxide dismutase; CAT, Catalase; MMP-2/9, Matrix metalloproteinase-2/9 (invasion markers); Iba1, Microglia/macrophage marker; HT-29, Human colorectal adenocarcinoma cell line; NAC, N-acetylcysteine (ROS, scavenger); n = 25, Sample size in human study.

Research found that Cd exposure resulted in more aggressive types of CRC cells by enhancing their ability to migrate and through activating pro-cancer signaling pathways in a CRC cell line (HT-29). Specifically, exposure to Cd results in an increase in cyclooxygenase-2 (COX-2) expression and an increase in prostaglandin E2 (PGE2) levels in an ROS-dependent manner *via* p38 MAPK signaling pathway activation, promoting CRC cell motility and invasion; increasing their ability to migrate implicates the Akt pathway in CRC, which is frequently altered in CRC. Inhibition of ROS or other signaling intermediates significantly reduced the Cd-induced migratory effects, indicating a mechanistic relationship between stress-induced signaling from Cd and the progression of CRC (Naji et al., 2019). In addition to causing immediate oxidative harm, Cd has very important effects on the epigenome, and these result in changes in the gene expression profile that can promote carcinogenicity.

Recent studies indicate that Cd may be a hypomethylating agent in colon carcinoma cells *via* downregulation of expression of DNA methyltransferases (DNMT) (Iftode et al., 2021). This could have a significant impact on the transcription of oncogenes and tumor suppressor genes. In addition to having documented associations with the effects of Cd on the epigenetics of human CRC cell lines at the level of global and gene-specific DNA methylation, Cd exposure is also associated with changes in global and gene-specific methylation states in numerous human CRC models. Thus, there is substantial indication that abnormalities in epigenetic regulation contribute to malignant transformation as a result of exposure to Cd. Furthermore, long-term epigenetic changes such as DNA methylation alterations and histone modification changes may compromise the integrity of the genome and aid in the survival of genetically unstable clones. Also, relevant experimental evidence indicates that epigenetic changes induced by Cd play an important role in the initiation and progression of carcinogenic processes separate from traditional paradigms of genetic mutation (Qu and Zheng, 2024).

The relationship between Cd exposure and key signaling pathways implicated in CRC further clarifies processes related to the formation of cancer cells. Cd exposure has been shown to interfere with the EGFR/Akt/mTOR axis, an important chain reaction needed for the growth and survival of CRC cells, providing additional means for tumor cells to spread through metastasis (Sun et al., 2023). The disruption of this pathway as a result of exposure to Cd emphasizes how environmental toxins can use and manipulate these signaling pathways involved in cancer growth and spread to further CRC progression. In addition, experimental data show that exposure to Cd disrupts cellular adhesion and the gut’s barrier function and thus may also facilitate tumor formation by facilitating paracrine signaling, evading attacks from the immune system, and interacting with pro-tumor microenvironments.

Although Cd’s role in CRC is now well recognized, there are major knowledge gaps that exist regarding the sequence of molecular events linking exposure to cancer and the subsequent tumor development. Examples include the dose-response relationship between Cd exposure and CRC carcinogenic risk, the levels of Cd exposure that have been demonstrated to be carcinogenic to human populations, and how other co-exposure toxicants may interact with the effects of Cd. Also, individual susceptibility, lifestyle (e.g., smoking, diet), and interactions between Cd and individual gut microbiota can potentially influence the carcinogenic properties of Cd. Recent studies have highlighted the need to incorporate toxic metal exposure into precision oncology principles in order to account for individual risk factors and responses to therapies (Yu et al., 2024). This growing evidence demonstrates that Cd has multiple roles in the process of CRC development. Cd destruction of colorectal epithelial cells occurs by oxidative stress generation, epigenetic modification, and creation of aberrant oncogenic signaling networks. Additional future research must be conducted to define the mechanisms of Cd (i.e., identify biomarkers of early carcinogenic exposure associated with Cd) and create preventative strategies to reduce overall cancer burden associated with environmental Cd. Understanding how Cd interacts with cellular and tumor micro environmental factors will guide public health policy and allow for individualized therapeutic interventions targeting the environmental causes of CRC.

## 4 Molecular overview of the Wnt/β-catenin signaling pathway

**FIGURE 1:**
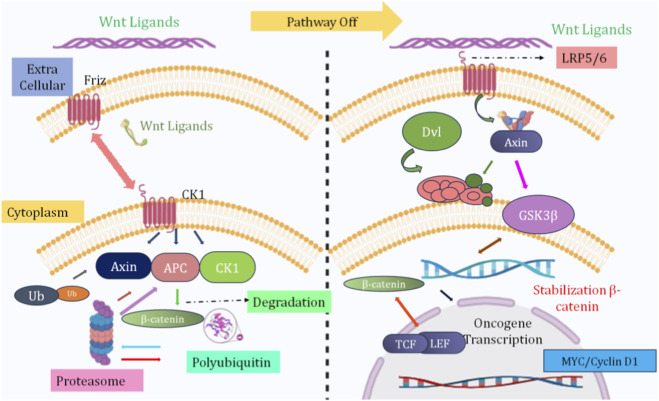
Molecular overview of the Wnt/β-catenin signaling pathway. In the absence of a Wnt ligand, β-catenin binds the destruction complex composed of APC, Axin, CK1, and GSK-3β. CK1 and GSK-3β phosphorylate β-catenin at N-terminal residues. Phosphorylated β-catenin undergoes ubiquitination and proteasomal degradation. Wnt ligand binding to Frizzled and LRP5/6 activates Dishevelled. The destruction complex becomes inactivated and dissociates. β-catenin stabilizes and accumulates in the cytoplasm. β-catenin translocates into the nucleus. Nuclear β-catenin binds TCF/LEF transcription factors. Target genes such as Cyclin D1, c-Myc, and AXIN2 are upregulated. Sustained pathway activation drives cell proliferation, survival, and tumor progression, including CRC.

The Wnt/β-catenin signaling pathway is an important molecular cascade that has existed throughout evolution and is vital for many processes during embryonic development, tissue maintenance (homeostasis), maintenance of stem cells, and differentiation of individual cells into different cell types (Figure 1). Dysregulation of the Wnt/β-catenin signaling cascade leads to a number of diseases, including cancers and fibrotic and metabolic diseases. Canonical Wnt signaling is primarily defined by Wnt’s ability to promote and maintain the stability of modulating the transcriptional activity of β-catenin, a protein that can link extracellular signals to the transcriptional response of target genes (Yu et al., 2024). Without Wnt ligands, the cytoplasmic concentration of β-catenin is strictly regulated by a multi-protein destruction complex made up of adenomatous polyposis coli (APC), axis inhibition protein (AXIN), glycogen synthase kinase 3 beta (GSK-3β), and casein kinase 1 alpha (CK1α).

Within this complex, β-catenin is sequentially phosphorylated by CK1α and GSK-3β, whereupon it is ubiquitinated for proteasomal degradation. These steps coordinate to prevent β-catenin from accumulating and inhibit the transcription of Wnt-responsive genes (Stamos and Weis, 2013). When Wnt ligands bind to Frizzled (FZD) receptors and low-density lipoprotein receptor-related proteins 5 or 6 (LRP5/6) co-receptors, canonical Wnt signaling begins. Binding of proteins causes the cytoplasmic scaffold protein Dishevelled (DVL) to be brought into the cytoplasm and triggers phosphorylation of LRP5/6 and the sequestering of AXIN at the plasma membrane. The destabilization of the destruction complex as the result of this event prevents phosphorylation and destruction of β-catenin, allowing for its stabilization and accumulation in the cytoplasm (Xue et al., 2025). When β-catenin has accumulated sufficiently in the cytoplasm, it then translocates to the nucleus and begins to interact with TCF/LEF transcription factors. Upon translocating to the nucleus, β-catenin sequesters the transcriptional co-repressors and recruits transcriptional co-activators (such as CBP/p300) to activate transcription of Wnt target genes involved in cellular proliferation, survival, and differentiation, including c-MYC, Cyclin D1, and AXIN2 (Wu et al., 2025).

The transcriptional actions of β-catenin are context-dependent and regulated by the cell type and developmental stage as well as other signaling pathways interacting with β-catenin signaling. Recent studies have suggested that post-translational modifications will play an important role in further fine-tuning Wnt and β-catenin signaling. The addition of phosphate, acetyl, ubiquitin, and methyl groups to Wnt/β-catenin pathway proteins modulates both the amplitude and duration of Wnt/β-catenin signals. In addition, E3 ligases located on membranes such as RNF43 and ZNRF3 inhibit Wnt signaling by mediating the degradation of Frizzled receptors, thus serving as an important negative feedback mechanism (Farnhammer et al., 2023; Tsukiyama, 2024). Also, the interaction of Wnt/β-catenin signaling with other pathways such as PI3K/AKT, Notch, and TGF-β will alter the cellular response to Wnt/β-catenin signaling, further contributing to the complexity of Wnt/β-catenin signaling. The Wnt/β-catenin signalling pathway is a highly regulated molecular system that is responsible for integrating extracellular cues with transcriptional regulation. The discovery of the molecular mechanisms behind the Wnt/β-catenin pathway has led to a greater understanding of disease pathogenesis and new therapeutic targets for Wnt-dependent diseases.

## 5 Epigenetic mechanisms in Cd-induced carcinogenesis

**FIGURE 2:**
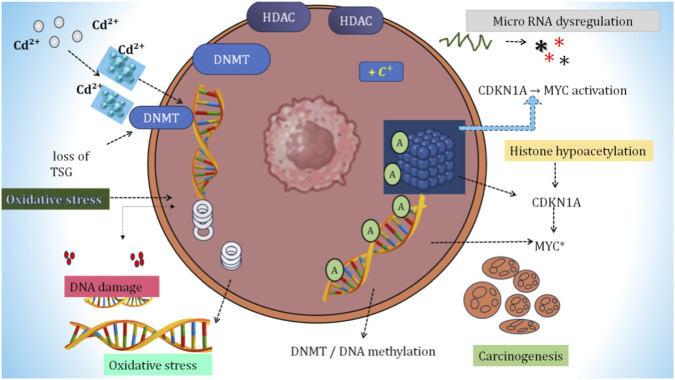
Molecular overview of the Wnt/β-catenin signaling pathway. The schematic illustrates how chronic Cd^2+^ exposure in intestinal epithelial cells drives CRC progression through epigenetic and signaling alterations. Cd^2+^ exposure enhances histone deacetylase and DNA methyltransferase activity, leading to widespread DNA hypermethylation and transcriptional silencing of key tumor suppressors, including APC, Axin, and CK1. Loss of these regulators disrupts the Wnt destruction complex, impairing β-catenin degradation. As a result, β-catenin accumulates in the cytoplasm and translocates to the nucleus, where it associates with TCF/LEF transcription factors to activate oncogenic targets such as Cyclin D1 and c-Myc. Sustained β-catenin signaling promotes CRC progression by enhancing cell proliferation, epithelial–mesenchymal transition, invasion, and genomic instability. Cd exposure further amplifies tumorigenic signaling through miR-31 upregulation, which augments PI3K/AKT pathway activity, reinforcing survival and growth signals.

Environmental toxicants, including Cd, have been shown to epigenetically regulate the canonical Wnt/β-catenin signaling pathway in a complex manner, creating an indirect link between exposure to environmental toxicants and oncogenic signal transduction (Figure 2). The disruption of the common epigenetic pathways occurs because of the disruption of specific patterns by cadmium and regulation of the structure of histones through changes in histone post-translational modifications and other non-coding RNA networks at each level of epigenetic regulation that intersects with critical nodes of the β-catenin pathway. The intersection of these multiple levels of epigenetic regulation is particularly demonstrated when examining the role of Cd in the carcinogenesis of many different cancers, including nasopharyngeal carcinoma (Zhao et al., 2022), triple-negative breast cancer (Kaleem et al., 2022), esophageal squamous cell carcinoma (Chen et al., 2022), and CRC (Sun et al., 2023), all of which show that the Cd-induced epigenetic reprogramming in these cancers functions like a genetic mutation in either APC, CTNNB1, or AXIN2 by consistently stabilizing nuclear β-catenin. Sharma et al. (2021) have established that epigenetic regulators play a role in modulating the transcription of the Wnt/β-catenin signaling pathway and also in regulating genes downstream of this pathway. Jiang et al. (2008) demonstrated that DACT3 acts as an epigenetically repressed antagonist to Wnt/β-catenin signaling within CRC, with Wnt/β-catenin activation being held in place by bivalent histones, whereas derepressing the Wnt/β-catenin pathway could induce apoptosis.

Epigenetic processes result in the activation of positive regulators of the WNT signaling pathways while simultaneously inactivating negative regulators. They can therefore influence the rate of cancer progression by contributing to the inappropriate activation of Wnt/β-catenin signal transduction pathways that, in turn, contribute to human cancer development, specifically colorectal cancer (Rajendran et al., 2024; Rajendran et al., 2026). Class I and Class III histone deacetylases (HDACs) have demonstrated the ability to epigenetically silence Wnt/β-catenin-regulated tumor suppressor genes (e.g., CDX1, EPHB) while not requiring mutations in the genes themselves (Rönsch et al., 2011). Chromatin regulators may also modulate the direct β-catenin-dependent transcriptional programs in CRC but have not yet been studied in the context of cadmium-associated epigenetic regulatory events (Bian et al., 2020). Qin et al. (2020) found that DNA methylation and histone modifications have a primary role in determining how colorectal cancer eventually develops. Postwala et al. (2023) found that the effects of the aforementioned epigenetic modifications and associated genetic alterations produce additional complexity in the regulation of the β-catenin signaling pathway. Boughanem et al. (2024) have shown that SFRP (secreted frizzled-related protein 2) gene silencing through early epigenetic mechanisms leads to active Wnt signaling for the duration of CRC development. Fully supporting previous conclusions, Wong and Pignatelli (2002) indicated that at all stages of CRC development, β-catenin serves as a critical regulator of the initiation of this malignancy.

## 6 Epigenetic regulation of the β-catenin pathway by Cd

A well-established and substantiated mechanism is promoter hypermethylation of Wnt antagonists that are expressed in the body. In nasopharyngeal carcinoma cells, treatment with CdCl_2_ at 1 μM concentration for several months leads to the hypermethylation of CpG islands in the casein kinase 1α (CSNK1A1) gene promoter region, which encodes for a protein that functions in the β-catenin destruction complex 1. The hypermethylation in this case refers to the increased addition of an abundant methyl group, that is, a carbon atom bonded to three hydrogen atoms. Therefore, the addition of more carbon enhances β-catenin accumulation and increases the level of β-catenin. A decrease in CSNK1A1 protein levels leads to reduced phosphorylation of β-catenin, which impairs its ubiquitination, and subsequent proteasomal degradation. As a result, β-catenin accumulates in the cell.

**FIGURE 3:**
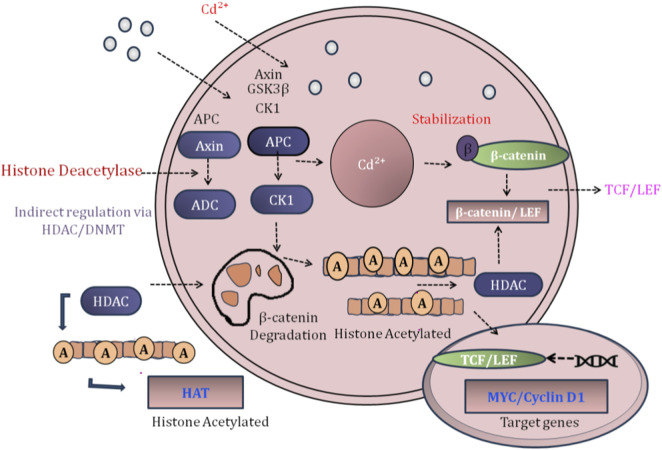
Cd-induced epigenetic dysregulation of Wnt/β-catenin signaling in CRC. Cd^2+^ exposure upregulates DNMTs and HDACs, leading to CpG island hypermethylation and silencing of tumor suppressor genes (APC, Axin, and CK1). Disruption of the Wnt destruction complex causes β-catenin accumulation and nuclear translocation, activating TCF/LEF-dependent transcription. Concurrent upregulation of miR-31 enhances PI3K/AKT signaling, reinforcing Wnt pathway activation and promoting CRC progression.

Through hypermethylation, CSNK1A1 is silenced. The hypermethylation of the CSNK1A1 promoter leads to a decrease in the amount of CSNK1A1 protein, resulting in an accumulation of β-catenin due to the inhibition of β-catenin phosphorylation, ubiquitination, and proteasome degradation of β-catenin. As a result, this leads to an accumulation of β-catenin in cells, translocation of β-catenin to the nucleus, and activation of the transcriptional activity of c-Myc, Cyclin D1, and survivin, which ultimately enhances cellular proliferation, migration, and chemoresistance. Likewise, exposure to Cd2+ has been linked to hypermethylation of the SFRP1 and Dkk1 promoters in other experimental models; SFRP1 and Dkk1 proteins are secreted frizzled-related proteins that sequester Wnt ligands or inhibit the activation of LRP5/6 co-receptors, which increases Wnt pathway activity by removing the upstream inhibitory signal (Figure 3) (Zhao et al., 2022; Wang et al., 2024).

While DNA methylation is not the only way Cd alters the histone epiproteome, it is one of the major means by which Cd alters the epiproteome in transformed human bronchial epithelial cells. Quantitative profiling by ELISA-based analysis of histone PTMs has shown widespread change globally in the amount of PTMs, including decreased levels of the H3K9ac mark of active enhancers and increased levels of H3K27me3, a Polycomb repressive mark at the locations of Wnt inhibitors (Liang et al., 2018). This occurs as part of a larger process of multiple levels of crosstalk between the histone epiproteome and DNA methylation machinery. For example, regions marked by H3K27me3 are preferential targets for *de novo* DNA methyltransferases DNMT3A/B, creating a self-sustaining silencing loop locking Wnt antagonist genes into a transcriptionally inactive state (Sharavanan et al., 2020; Olmedo-Suárez et al., 2022). In addition, Cd induces an increase in the expression of EZH2, which is the catalytic subunit of PRC2, which catalyzes the addition of H3K27me3 in hepatocellular carcinoma cells (Ghosh et al., 2020). This increase in EZH2 expression is driven by epigenetic mechanisms, as Cd induces a CpG demethylation of the EZH2 promoter and, therefore, forms a feed-forward, oncogenic circuit in which Cd initiates and amplifies its own epigenetic dysregulation.

The non-coding RNAs are the third type of Cd-mediated β-catenin regulatory mechanism and represent an evolving and complex mechanism. Exposure to Cd results in the modification of many miRNAs and long non-coding RNAs targeted toward important components of the pathway. In triple-negative breast cancer cells, Cd induces an increase in miR-200c and subsequently inhibits ZEB1, a transcriptional repressor of E-cadherin 2. While this would suggest that the result is anti-metastatic, it is complicated by the fact that ZEB1 also represses DVL2, a known positive regulator of Wnt signaling, which means that miR-200c-mediated ZEB1 downregulation indirectly augments DVL2 expression and subsequently potentiates beta-catenin stabilization. Additionally, Cd induces an increase in circ_000999, a circular RNA that acts as a molecular sponge for miR-205-5p (Wang et al., 2024). miR-205-5p normally targets and degrades ZEB1 mRNA; therefore, sequestration of miR-205-5p by circ_000999 allows for ZEB1 accumulation, which promotes epithelial mesenchymal transition (EMT) and leads to nuclear β-catenin translocation, an indication of metastatic progression. This very complex interaction between RNAs is an example of how Cd uses the inherent logic of ncRNAs to amplify oncogenic signaling pathways.

Epigenetic reprogramming has functional biological and clinical consequences. For example, *in vivo* studies of mice exposed to Cd demonstrated greater tumor burden and faster metastasis to lung and breast tissues; these phenotypes could be reversed through pharmacological inhibition of β-catenin *via* the administration of demethylating agents (e.g., 5-aza-2′-deoxycytidine) (Peng et al., 2018; Li et al., 2024). Additionally, there are strongly positive correlations between Cd levels as measured by blood or urine from epidemiologic studies conducted in geographic areas with high levels of exposure and specific types of Wnt/β-catenin-driven cancers, the incidence, stage, and lymph node metastasis of which can largely be predicted based on the levels of Cd in those areas (Peng et al., 2018; Chen et al., 2022).

Importantly, these effects occurred at environmentally relevant concentrations (<1 µM CdCl_2_ *in vitro* or <3.6 mg/L Cd in the drinking water of the relevant study population of 112 mice), indicating that Cd can promote cancer development *via* non-genotoxic pathways. Rather than functioning as a classic genotoxic carcinogen that induces tumors through direct damage to DNA, Cd functions as an epigenetic carcinogen that co-opts the Wnt/β-catenin cell signaling pathway to elicit disease. This integration of findings leads to a single conceptualization: Cd, through zinc transporters such as ZIP8 and ZIP14 (Satarug et al., 2021), is taken into cells; it causes oxidative stress and interrupts one-carbon metabolism (Qu and Zheng, 2024), all of which lead to disruptions in the fidelity of epigenetic writer and eraser enzymes (e.g., DNMTs, TETs, HDACs, HATs, and EZH2). This overall epigenetic instability then becomes channeled to the Wnt/β-catenin pathway, resulting in a persistent level of oncogenic signal *via* initial silencing of negative and then activation of positive regulators. This model is why Cd is considered a Group 1 carcinogen by the International Agency for Research on Cancer (IARC) despite being considered non-mutagenic (Zhang et al., 2022; Hussein et al., 2024).

The cancer-causing ability of Cd is caused not only by apoptosis but also through epigenetic alteration of the cellular signals associated with the DNA strand. As a result, the future of cancer therapy will include using non-cytotoxic agents to target the future uses for epigenetic targeting of these cancer-causing signals. For example, combining β-catenin inhibitors with the epigenetic modulating agents used in this study can interrupt the self-sustaining cycle of Cd-induced oncogenesis. The accumulation of β-catenin as a result of Cd can occur through the epigenetic silencing of components of the destruction complex and the signal transduction pathway that activate it. For a detailed explanation of how Cd causes β-catenin accumulation through this epigenetic process, see Table 2. This epigenetic alteration of DNA sequences activates c-Myc and cyclins and causes the development of the key features of cancer. These epigenetic alterations can be reversed through the use of agents that demethylate DNA, through gene silencing approaches such as RNA interference, or through using agents that block the effects of epigenetic alterations at the level of protein synthesis dilatory interventions.

**TABLE 2:** Cd-driven epigenetic activation of Wnt/β-catenin pathway.

Epigenetic mark	Target gene/Component	Cd effect	β-Catenin impact	Cancer type/Model	Key markers/Pathways affected	Evidence type	Dose/Duration	Reversal method	Functional outcome	References
DNA hypermethylation	CK1α promoter	↑ methylation (5 mC↑)	Stabilizes β-catenin (↑ nuclear)	NPC cells (CNE1/2)	Wnt targets: Cyclin D1/E, c-Myc↑	*In vitro*/chronic	1μM Cd, 10 weeks	5-Aza-CdR restores CK1α	↑ proliferation, EMT, invasion	Zhang et al., 2024; Peng et al., 2018
Histone H3K9 demethylation	AXIN2, DKK1 promoters	KDM3↑ erases H3K9me2	↑ β-catenin/Tcf transcription	CRC stem cells (ALDH+)	H3K4me (MLL1), PYGO2/BCL9 recruit	*In vitro*/xenograft	N/A (KDM3 KD)	KDM3A/B depletion	↓ Tumorigenesis, CSC survival	Li et al. (2017)
DNA hypermethylation	Wnt inhibitors (SFRP5)	↑ CpG methylation	↑ β-catenin signaling	Colon cancer (AOM/DSS)	H3Ac↓ at promoter	Animal/dietary GEN	Chronic low-dose	GEN diet induces methylation	↓ Wnt hyperactivation	Yang et al., 2025; Alqalalwah et al., 2025
Histone deacetylation (SIRT1)	β-catenin locus	SIRT1↑ deacetylates H3K9/14	↓ Active marks, heterochromatin	General cancer (B cells)	H3Ac↑ upon SIRT1↓	Review/*in vitro*	Cd mimetic HDACi	NAD+ deprivation	↑ Gene expression, proliferation	Gan et al. (2020)
miRNA dysregulation	CTNNB1 (β-catenin)	miR-142-3p↓	↑ β-catenin protein/nuclear	CRC cells (HT29/HCT116)	Wnt/β-catenin activation	*In vitro*/overexpression	N/A (miR mimic)	miR-142-3p OE	↓ Tumorigenesis	Liu et al. (2021)
Global hypomethylation	Wnt pathway genes	Global 5 mC↓, DNMT↑	Aberrant activation	Prostate/CRC models	APC/Axin/β-catenin epigenome	Review/epidemiology	Environmental Cd	Demethylase inhibitors	↑ Oncogene expression	Sharma et al. (2021)

## 7 Role of Cd-induced epigenetic alterations in CRC progression

**FIGURE 4:**
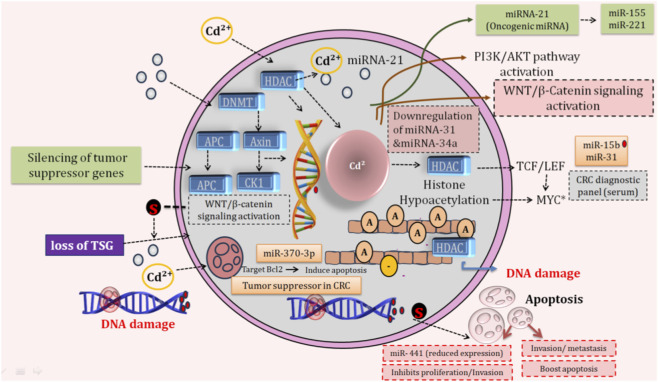
The figure depicts multi-layer epigenetic disruption induced by cadmium (Cd2+) in a colorectal epithelial cell. Cd2+ enters the cell and translocates to the nucleus, where it increases the activity of histone deacetylases and DNA methyltransferases. This leads to DNA hypermethylation and transcriptional silencing of tumor suppressor genes, including APC, Axin, and CK1. Loss of these regulators disrupts the Wnt destruction complex in the cytoplasm, resulting in β-catenin stabilization and accumulation. Stabilized β-catenin translocates to the nucleus and interacts with TCF/LEF transcription factors, activating oncogenic targets such as Cyclin D1 and c-Myc. These events promote colorectal cancer progression through enhanced cell proliferation, epithelial-mesenchymal transition, and invasive capacity. Cadmium exposure further amplifies tumorigenic signaling *via* miRNA dysregulation. miR-31 is upregulated and enhances PI3K/AKT signaling, supporting cell survival and growth. miR-21 functions as an oncomiR by suppressing additional tumor-suppressor pathways. In contrast, miR-34a, a negative regulator of Wnt signaling, is suppressed. Together, these epigenetic and post-transcriptional changes lead to persistent DNA damage and maintenance of a pro-tumorigenic cellular state.

Exposure to Cd is thought to affect the progression of CRC through the regulation of microRNA expression, oxidative-reductive (redox) balance, and β-catenin signaling (Figure 4). Liu et al. (2021) previously showed that miR-142-3p directly targets the mRNA of β-catenin and decreases the concentration of β-catenin in the nucleus of CRC cells, thereby decreasing the proliferative capacity of CRC cells and indirectly decreasing the production of ROS by inhibiting Wnt signaling. There are also several miRs whose expression is regulated by Cd and promote apoptosis *via* the induction of oxidative stress. For example, the expression of miR-370-3p and miR-124-3p is enhanced in Cd-exposed intestinal cells, hence inducing apoptosis through the regulation of the anti-apoptotic protein Bcl-2 when cells are subjected to oxidative stress (Yang et al., 2021; Lei et al., 2020). Lei et al. (2020) also demonstrated that miR-122-5p and miR-326-3p regulated pathways associated with phospholipase D1 (PLD1)-mediated apoptosis and oxidative stress in renal models exposed to Cd, demonstrating that there are conserved Cd-induced miR responses across different tissues.

The exposure to Cd was found to activate miRNA programs that promote oxidative stress by accumulating ROS and activating Wnt signaling pathways. For example, Khalilian et al. (2023) and Lian et al. (2023) found that miR-210 is overexpressed in hypoxic and mitochondrial-stressed environments. This miR-210 expression was associated with increasing levels of ROS and poor overall survival in patients with colon cancer. Beni et al. (2022) found that miR-224 enhances the activation of the Wnt/β-catenin pathway in colon cancer by inhibiting GSK3β (glycogen synthase kinase 3 beta) and SFRP2 (secreted frizzled-related protein 2), stabilizing β-catenin. Lin et al. (2017) found that the oncomiR miR-21 in the context of Cd exposure promotes ROS (by inhibiting SOD2 and SOD3) while also activating the PTEN/PI3K/AKT/β-catenin signaling pathway. Previous studies found that the tumor suppressor miR-34a inhibits the action of ROS scavenging enzymes and reduces the expression of Wnt target genes Axin2 and Snail1 in colon cancer cells, sensitizing these cells to undergo apoptosis when exposed to high levels of oxidative stress (Gao et al., 2024).

MiRNAs associated with inflammation increase the connections between Cd toxicity and the activation of redox and epigenetic signaling pathways. Lei et al. (2020) identified raised levels of miR-221, miR-222-3p, miR-30c, and miR-155 in tissues exposed to Cd; the authors linked these miRNAs to inflammatory signaling and to the possible amplification of ROS. Lu et al. (2020) show that miR-212 inhibits manganese superoxide dismutase, thus stimulating ROS-mediated metastasis, and that miR-155 inhibits the activity of the oxidative stress enzymes SOD2 and catalase in cancers that are resistant to chemotherapy. Ghuwalewala et al. (2021) showed that miR-146a stimulated AKT and β-catenin signaling, thus supporting tumor stemness maintenance in these cancers due to the effects of Cd. These findings suggest that Cd-induced epigenetic reprogramming *via* miRNAs results in ongoing oxidative stress, stabilization of β-catenin signaling, and support for the proliferation, invasion, and stemness phenotype of colorectal tumors. Additionally, Cd exposure leads to the alteration of miRNA profiles; therefore, miRNAs promote apoptosis (miR-370 or -124) or inflammation (miR-21, -155, or −221 in intestinal or renal epithelial cell models). Finally, there is significant overlap between those oncogenic miRNAs that are dysregulated within the plasma and tissues of colorectal cancer patients (miR-21, -146a, and −155) and those miRNAs that are dissociated from the development of colorectal cancer (Table 3).

**TABLE 3:** miRNA regulation across Cd toxicity and CRC contexts.

Study focus	Key miRNAs	Findings	Source
Cd in IEC-6 cells	miR-370-3p, miR-124-3p	Target Bcl-2 to induce apoptosis in intestinal cells	Yang et al. (2021)
Cd toxicity review	miR-21, miR-155, miR-221	Upregulated in Cd-exposed tissues/organs, linked to immune changes	Koushki et al. (2024)
CRC miRNA panel	miR-29a, miR-101, miR-125b, miR-146a, miR-155	Dysregulated in CRC vs. healthy tissue, tied to proliferation	Ždralević et al. (2023)
Cd in kidney cells (NRK-52E)	miR-122-5p, miR-326-3p	Enhance Cd-induced apoptosis by downregulating PLD1	Koushki et al. (2024)
Cd in rat renal cortex	44 upregulated, 54 downregulated miRNAs	Alters miRNA profile in Cd nephrotoxicity	Koushki et al. (2024)
Cd occupational exposure	miR-363-3p	Increased in serum; tumor suppressor in CRC and other cancers	Koushki et al. (2024)
CRC cell lines/tissues	miR-4461	Reduced expression inhibits proliferation, migration, invasion	Shirzad et al. (2025)
CRC tissues	miR-200a, miR-21	Elevated; suppression reduces invasion and boosts apoptosis	Shirzad et al. (2025)
CRC diagnostic panels	miR-15b, miR-21, miR-31	High accuracy for early CRC detection in serum	Sur et al. (2022)

## 8 Epigenetic basis of chemoresistance in Cd-exposed CRC

**FIGURE 5:**
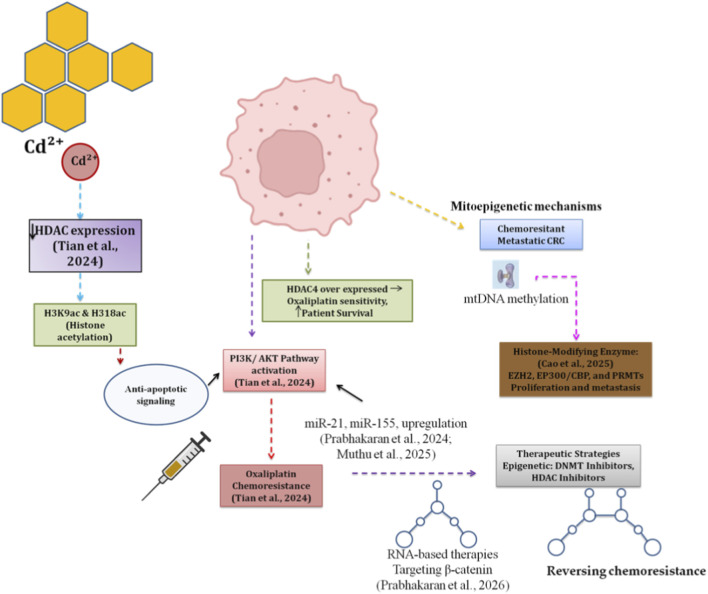
Epigenetic basis of chemoresistance in Cd-exposed CRC.

Tian et al. (2025) revealed new evidence for a previously unrecognized mechanism whereby TGF-β pathway activation leads to decreased expression of HDAC4, resulting in increased levels of both H3K9ac and H3K18ac. The histone acetylation encountered in this cascade subsequently induces oxaliplatin chemoresistance by triggering the anti-apoptotic PI3K/AKT signaling pathway. CRC cells showed a significantly greater sensitivity towards oxaliplatin chemotherapy when HDAC4 was overexpressed, and HDAC4 expression was positively correlated with patient survival. In contrast, Cao et al. (2025) highlighted the emerging role of mitoepigenetic mechanisms as playing a critical role in the development of chemoresistant metastatic colorectal cancer. Furthermore, histone-modifying enzymes such as EZH2, EP300/CBP, and PRMTs were shown to be important drivers of colorectal tumorigenesis due to their ability to alter the transcriptional program specific to cell proliferation and metastasis. Inhibitors have already begun providing new avenues for treating cancer patients *via* the use of epigenetic treatment strategies. Ma et al. (2023) reviewed the latest strategies aimed at reversing chemoresistance, including novel strategies based upon epigenetic therapy. Hence, DNA methyltransferase inhibitors and other types of epigenetic agents represent promising candidates for overcoming drug resistance in the treatment of CRC (Figure 5).

### 8.1 Clinical implications

These recent studies provide evidence that epigenetic mechanisms are now viewed as potentially reversible and dynamic targets for therapeutic intervention. The identification of histone modifications and mitochondrial epigenetic changes also provides potential precision therapeutic options for treating chemoresistance in colorectal cancer. No studies reviewed have examined how cadmium exposure affects the mechanisms of epigenetic chemoresistance, meaning this is still an unknown field in need of further study.

## 9 Therapeutic and preventive perspectives

There is an increasing recognition that the epigenetic dysregulation of the β-catenin signaling pathway contributes to Cd-induced CRC, leading to new opportunities for treatment or prevention of these diseases. An exposure to Cd has been shown to activate β-catenin signaling through epigenetic processes, such as changes in DNA methylation, modifications of histones, and disruption of the regulation of non-coding RNAs, all of which act together to facilitate tumor development, increase stemness, and ultimately induce chemoresistance (Zeng et al., 2025; Hu et al., 2026). The use of therapies directed at reversing these epigenetic alterations may represent a novel method of blocking Cd-driven oncogenic signaling. Therapies based on epigenetics, like inhibitors of DNMTs or of histone deacetylases, have been recognized as being able to reverse abnormal gene expression patterns related to the regulation of β-catenin.

In models of CRC, DNMT inhibitors (e.g., azacitidine and decitabine) have been shown to reactivate silenced antagonists of Wnt signaling, such as DKK1 and members of the SFRP family, leading to a reduction in β-catenin-mediated transcriptional activity (Gupta, 2025). In the same manner, HDAC inhibitors can alter the accessibility of chromatin at the promoter regions of β-catenin target genes, leading to a decrease in CRC proliferation and increased sensitivity of CRC to chemotherapy (Guan et al., 2024). Targeting the epigenetically regulated non-coding RNAs (ncRNAs) that are involved with β-catenin signaling is a good approach for developing new ways to therapeutically intervene in disease. Two examples of such microRNAs are miR-34a and the miR-200 family members, which are often silenced due to promoters becoming hypermethylated by Cd exposure. These microRNAs function as negative regulators of β-catenin signaling and epithelial–mesenchymal transition (EMT).

Studies have demonstrated that restoration of these microRNAs decreases damage caused by CRC cells migrating from the primary tumor and decreases the ability to become resistant to chemotherapy treatment (Wu et al., 2018). In addition, research is beginning to identify long noncoding RNAs and circRNAs that may be formed due to Cd exposure, which could provide new potential treatment targets for precision approaches to CRC therapy. Reducing environmental and occupational Cd exposure continues to be an important public health concern due to its potential for causing disease. Some studies have shown that dietary interventions with natural epigenetic receptor modulators such as polyphenols, flavonoids, and omega-3 fatty acids provide a protective effect against both Cd-induced epigenetic reprogramming and β-catenin activation (Abbas et al., 2021).

These bioactive compounds can affect how DNA is methylated and histones are acetylated, which will assist in keeping normal Wnt/β-catenin signal homeostasis. The ability to quickly identify epigenetic biomarkers related to cadmium exposure, for example, methylation profiles of Wnt pathway-regulating proteins, may help in assessing an individual’s level of risk for Cd-related health problems and developing appropriate chemopreventive interventions. By combining the use of therapeutic epigenetics with traditional chemotherapy, the success rate of treatment may increase, and β- Catenin-associated chemoresistance could become less problematic. Overall, therapeutically targeting and preventing the modulation of the β-catenin genome will provide a highly innovative and clinically applicable way to prevent cadmium exposure from leading to the development of CRC.

Several newly developed therapies involving epigenetic modifications to chromosomes provide potential avenues for reducing the abnormal activation of Wnt signaling through alterations to DNA methylation and histone acetylation in colorectal cancer. For example, the use of DNA methyltransferase inhibitors (DNMTIs), including decitabine and 5-aza-2′-deoxycytidine (5-AzaDC), can inhibit activation of the Wnt signaling crucial for CRC development by reversing the hypermethylation of the promoters of Wnt antagonists. According to Peng et al. (2025), decitabine reduces the activity of Wnt inhibitors by facilitating the ubiquitin-mediated degradation of DNMT1 and EZH2, thereby restoring tumor suppressor gene expression and ultimately inhibiting CRC development. The drug 5-azacytidine (5-AzaDC), like the drug decitabine, can reactivate certain Wnt-inhibiting proteins that were previously silenced, such as SFRP1, thus leading to the loss of cancer stem cell characteristics and slowing tumor growth (Li et al., 2018). Consequently, it is reasonable to believe that the DNMTi class of drugs will reduce tumor growth by modifying Wnt pathways *via* epigenetic changes.

CRC also uses histone deacetylase inhibitors (HDACis) to regulate Wnt/β-catenin signaling *via* both histone and non-histone pathways. An example is Vorinostat (SAHA), which is an FDA-approved HDAC inhibitor that works by promoting the proteasome-mediated degradation of TCF7L2, a transcriptional effector of the Wnt signaling pathway, thereby promoting decreased activation of the pathway and increased apoptosis of CRC cells (Bates, 2020). Among the HDAC inhibitors developed, ZDLT-1 appears to induce apoptosis *via* two different pathways (HIF-1alpha and caspase) and may also indirectly alter how the Wnt signaling pathways work (Geng et al., 2024). Hydroxamate-based HDAC inhibitors may also change Wnt signaling from canonical to non-canonical pathways, causing different cellular responses and, thus, providing possible therapeutic benefits. Recent studies have demonstrated that dual DNMT/HDAC inhibitors and combination approaches may be effective at targeting Wnt-driven CRC. CO2S and (R)-23a are both dual inhibitors that can alter the methylation of DNA as well as histone acetylation since both changes are mediated through the β-catenin pathway and will activate local anti-tumor immune responses (Yang et al., 2024; Chang et al., 2024). The aforementioned compounds are capable of altering tumor immune microenvironments by activating interferon signaling and enhancing responses to immune checkpoint inhibitors (e.g., anti-PD-L1 therapy).

Beyond just inducing apoptosis and overcoming resistance to therapy, the combination of DNMT and HDAC inhibition through agents like guadecitabine (DNMT1 inhibitor) and vorinostat (HDAC inhibitor) has already been shown to reactivate Wnt antagonists that had previously been silenced, thus enhancing the efficacy of the immune system-mediated tumorigenic suppression (Li et al., 2024). The goal of combining HDAC inhibitors with targeted therapies such as ABT-199 is to induce apoptosis in cancer cells and overcome the development of resistances in patients who have received a diagnosis of metastatic colorectal cancer (Abu Alsamen et al., 2025). Ultimately, advanced technologies such as CRISPR-based epigenetic modulations will allow for direct modifications to enhance or limit transcriptional activity along the Wnt/β-catenin transcriptional regulation pathway. Targeted chromatin modifications at β-catenin-associated sites may represent an alternative means of inhibiting excessive functioning of the oncogenic CTCR (Huang et al., 2024). Targeted approaches that modify histones with various acylation, methylation, and/or phosphorylation changes are being investigated as potential ways to alter chromatin structures and reduce Wnt signaling in different subtypes of CRC (Jaafari, 2026). These advances create an increasing number of opportunities to explore epigenetic therapeutics for CRC and the substantial ability of epigenetic therapies targeting the Wnt/β-catenin pathway.

## 10 Discussion

There is increasing evidence that exposure to Cd in the environment causes CRC by inducing oxidative stress, causing chronic inflammation, epigenetic dysregulation, and activating oncogenic pathways (Genchi et al., 2020; Chen et al., 2019). Moreover, epigenetic changes induced by Cd have been shown to activate the Wnt/β-catenin signaling pathway, which may also promote CRC progression and resistance to chemotherapy (Sharma et al., 2021; Zhang and Wang, 2020). Cd, an environmental chemical, can alter the epigenome by changing DNA methylation patterns and histone modifications as well as by changing expression levels of ncRNAs. These alterations will result in the subsequent silencing of tumor suppressor genes, leading to tumor development. As a result, the epigenetically altered cells have the capacity to modify molecules associated with the Wnt/β-Catenin cascade, thereby changing Wnt/β-Catenin signaling mediated *via* modifications in key regulatory proteins of the Wnt/β-Catenin signaling cascade; for example, by increasing levels of β-Catenin and increasing the expression of oncogenic target genes (e.g., c-Myc and Cyclin D1) (Song et al., 2024). Sustained activation of Wnt/β-catenin promotes the survival of cancer stem cells, induces epithelial mesenchymal transition (EMT) and enhances the mechanisms of drug efflux (Zhang and Wang, 2020) thus contributing to the development of chemotherapeutic resistance in patients. Dysregulation of microRNAs induced by Cd exposure may contribute further to this outcome by maintaining oncogenesis signaling pathways and enhancing the resistance of patients to therapy (Beni et al., 2022; Koushki et al., 2024); while there have been advances in our understanding of the relationship between Cd exposure, epigenetics and Wnt signaling, however, the specific mechanism by which Cd exposure affects epigenetic modification and the regulation of Wnt signaling *via* epigenetic modifications and the regulation of Wnt signaling remain to be elucidated. Future studies should address the identification of epigenetic biomarkers as well as regulatory networks associated with these biomarkers that will be instrumental in understanding how Cd affects cancer. There are several epigenetic regulators of the Wnt/β-catenin signalling pathway that are targeted for future research to overcome chemoresistance through the development of miRNA-based therapies and DNMT/HDAC inhibitors (Geng et al., 2024; Peng et al., 2025). Overall, these data support the view that Cd-induced epigenetic dysregulation is an important mechanism that links environmental exposures to Wnt/β-catenin driven CRC progression and resistance to chemotherapy, thus requiring further investigation into both the mechanism as well as potential treatment options for the disease.

## 11 Knowledge gaps and future research directions

There is increasing evidence that cadmium exposure might cause the abnormal activation of the β-catenin signaling pathway through epigenetic mechanisms in CRC, yet several key knowledge gaps still exist, limiting translational and clinical progress. One of the most significant obstacles to progress is the lack of an understanding of how chronic, low-dose exposure to Cd, which better represents environmental and dietary exposures, may lead to stable epigenetic modifications in the genes that regulate β-catenin over time. The bulk of the existing studies have been conducted using acute or high-dose exposure models, which may not truly reflect the type of exposures in the real world (Peng et al., 2018). The second major issue is the epigenetic hierarchy regulating β-catenin dysregulation due to Cd exposure and its role in Cd-related CRC. There is evidence implicating DNA methylation, histone modifications, and non-coding RNAs; however, we still do not understand how they work together or in what order they do so during the initiation and progression of tumors. Moreover, we do not know whether cadmium-induced hypermethylation of Wnt antagonist genes comes before the remodelling of histones or if non-coding RNAs are major reasons to drive β-catenin signalling or serve to modulate it (Qian et al., 2025).

Closing this gap requires the development of integrative multi-omics strategies that combine epigenomics, transcriptomics, and proteomics. Research into epigenetic diversity in the tumor microenvironment is still in its early days. Recent studies show that exposure to Cd might affect how cancer-stem cells, immune cells, and stromal cells are altered by Cd, resulting in β-catenin-mediated chemoresistance and evasion of the immune system (Ghuwalewala et al., 2021; Hu et al., 2026). However, there are very few spatial and single-cell studies of epigenetic variation in Cd-induced CRC. There is a critical need to explore epigenetic variability at the single-cell level in order to identify therapeutic targets that are specific to individual cell types. There is little clinical evidence supporting the use of epigenetic biomarkers associated with Cd exposure and activated β-catenin from a therapeutic perspective. Many candidate methylation signatures and ncRNAs have been identified, but very few have demonstrated any predictive value regarding resistance to chemotherapy or sensitivity to treatment in a patient population (He et al., 2022). Future research should prioritize longitudinal clinical study designs that produce clinically meaningful epigenetic biomarkers that can be used to predict an individual’s risk for developing the disease, how well they are likely to respond to treatment, and how well they will do overall after being treated.

Preventive research is still an area that has not been fully explored. Zhao et al. (2025) have stated that while there are dietary epigenetic modulators and lifestyle practices that have been shown to have promise in reducing the effects of epigenetic reprogramming from Cd on the host, there are not many mechanistic or population-based studies looking at these two methods of prevention. Future research should include integration of environmental exposure assessments and epigenetic risk modeling, which can be used to help inform public health strategies. Overall, closing these knowledge gaps will be critical to be able to develop and advance precision prevention and epigenetic-based therapies aimed at β-catenin signaling in patients with colon cancer associated with Cd exposure.

## 12 Conclusions and future perspectives

The mounting evidence indicates that epigenetic dysregulation significantly contributes to the development of β-catenin-driven CRC from exposure to Cd and the development of chemoresistance. This review shows that Cd should be considered not only as a harmful environmental contaminant but also as a powerful epigenetic modulator, with the ability to change key regulatory nodes of the Wnt/β-catenin signaling pathway. Cd reprograms the transcriptional regulation of β-catenin and its downstream oncogenic targets by altering DNA methylation, histone modifications, and non-coding RNA expression. Ultimately, the reprogramming of the transcriptional control of β-catenin and its oncogenic targets promotes the initiation of tumors, continued proliferation, the expression of cancer stem cell characteristics, and the development of resistance to traditional chemotherapy. The reversible characteristic of epigenetic alterations provides an opportunity to find a way to treat cancer. The epigenetic silencing of Wnt inhibitors and changes to chromatin at genes under the control of β-catenin along with non-coding RNA that is not functioning correctly contribute to long-term activation of pathways related to CRC caused by Cd. Additionally, these changes will either create a larger opportunity for cancer cells to grow or create a greater risk that the treatments will not work, which supports the need to combine epigenetic modulators with standard chemotherapy techniques to produce an integrated treatment approach.

Epigenetic markers related to cadmium exposure and β-catenin activity show promise in helping to identify CRC earlier, classify patients according to their risk for CRC, and create individualized treatments. There is still a large gap between mechanistic knowledge gained from research and the translation of that knowledge into actual use in the clinic. Longitudinal and population-based research should be prioritized to better elucidate the development of epigenetic signatures specific to exposures and the function of how those signatures influence wellness. Additional multi-omic and single-cell approaches to determine tumor heterogeneity, as well as environment-specific epigenetic regulation of β-catenin, are critical to informing future studies. Combining environmental monitoring with epigenetic interventions through diet and lifestyle may lead to innovative and sustainable methods of reducing CRC risk associated with cadmium exposure. Ultimately, understanding how environmental carcinogens affect epigenetic regulation of the β-catenin pathway will be a critical dynamic for creating therapeutic and preventive options for CRC.
